# Coping strategies, vision-related quality of life, and emotional health in managing retinitis pigmentosa: a survey study

**DOI:** 10.1186/s12886-018-0689-2

**Published:** 2018-01-30

**Authors:** Krithika Anil, Gulcan Garip

**Affiliations:** 1 University of Derby Online Learning, Derby, England, United Kingdom; 2 University of Derby, Kedleston Road, Derby, England, DE22 1GB United Kingdom

**Keywords:** Retinitis pigmentosa, Coping, Vision-related quality of life, Emotion, Home

## Abstract

**Background:**

Retinitis pigmentosa is a group of genetic progressive retinal dystrophies that may adversely affect daily life. Those with RP should develop adaptive coping strategies to manage their condition. This study investigates the relationship between engaging (ECS) and disengaging coping strategies (DCS), vision-related quality of life (VRQoL), and emotional health, in adults living at home with retinitis pigmentosa.

**Method:**

One hundred and five participants (70 female; mean_age_ of 46.98, *SD*_*age*_ = 13.77) completed a cross-sectional survey. The questionnaire booklet consisted of the Coping Strategies Inventory – Short Form (32 items), the National Eye Institute Visual Functioning Questionnaire 25 (25 items), Marylands Trait Depression Scale (18 items), the Warwick-Edinburgh Mental Well-being Scale (14 items), and the Subjective Happiness Scale (4 items).

**Results:**

Data was analysed with a two-block hierarchical multiple regression, with the first block controlling for the demographic data (age, sex, years since retinitis pigmentosa diagnosis, number of comorbidities, participant-perceived retinitis pigmentosa severity, and knowing RP type) and the second block consisting of primary measures (type of coping strategy, VRQoL, and Emotional Health). Type of coping strategy was found to impact psychosocial variables of VRQoL, not overall VRQoL. These psychosocial VRQoL variables had a positive association with ECS and a negative association with DCS. Emotional Health increased with ECS and decreased with DCS. There was a larger impact of DCS on VRQoL and Emotional Health compared to ECS, that is, VRQoL and Emotional Health decreased more with increasing DCS than VRQoL, and Emotional Health increased with increasing ECS.

**Conclusion:**

In concordance with previous research, ECS increased with increasing VRQoL and DCS decreased with increasing VRQoL. However, the findings also indicated that DCS had a greater impact than ECS on VRQoL and Emotional Health. This suggests that diminishing DCS should be prioritised over developing ECS to positively influence VRQoL and Emotional Health. Further research should investigate the impact of reducing DCS compared to increasing ECS, and how this may influence VRQoL and Emotional Health.

**Electronic supplementary material:**

The online version of this article (10.1186/s12886-018-0689-2) contains supplementary material, which is available to authorized users.

## Background

Retinitis pigmentosa (RP) is a group of genetic progressive retinal dystrophies that have distinct symptoms, such as impaired night vision and gradual loss of peripheral vision [1]. RP-related visual impairments negatively impact the performance of individuals’ daily activities, such as driving, cooking, and self-grooming [2]. The ability to effectively complete daily activities gradually decreases and eventually plateaus as the RP progresses to its final stage [2]. This leads to a decrease in vision-related quality of life (VRQoL), which is the extent to which vision impacts life satisfaction and the ability to complete daily activities [2]. There is currently no cure for RP [1], and its prevalence is estimated to be 1 in 4000 in the UK [3]. It is vital to assess the VRQoL in those living with RP due to the negative effect RP may have on everyday life.

Individuals with RP become more dependent on others for daily tasks, which can improve their support network [4, 5]. However, being unable to independently complete previously achievable tasks has been reported to cause frustration and low levels of self-confidence, leading to dissatisfaction with life, and the development of depressive symptoms [2, 6–8]. Thus, individuals with RP report reduced emotional health. Reduction in emotional health lowers their incentive to continue normally with their daily tasks and stops individuals from pursuing solutions to their vision-related problems [9]. Thus, VRQoL is likely a key factor influencing emotional health.

Different coping strategies, such as ignoring the problem or confiding in others, may influence an individual’s adaptation to vision impairment; this adaptation is associated with VRQoL [10, 11]. Sturrock and others [9] state that acceptance coping (a type of engaging coping strategy) was associated with better adaptation to vision impairment and that disengaging coping strategies (ignoring or withdrawing from the situation at hand), was associated with worse adaptation to vision impairment. As coping strategies affect how an individual may deal with their vision-related problems, coping strategies may also influence their ability to complete daily tasks [6], thus influencing VRQoL.

Coping strategies and emotional health may be associated with each other. Sturrock and others [9] state that there is a maladaptive cycle of disengaging coping strategies leading to lower emotional health, which leads to the further use of disengaging coping strategies. This suggests a relationship between emotional health and coping strategies, where engaging or disengaging coping strategies may indicate emotional health levels. Being diagnosed with RP is associated with developing depressive symptoms, poor mental wellbeing, and lower levels of general happiness with life [7]. Therefore, coping strategies and emotional health seems to be highly associated with each other.

Current published literature does not examine the relationship between coping strategies, VRQoL, and emotional health in the population of individuals with RP in their daily lives. This should be investigated because RP is a life stressor that demands individuals to constantly adapt to daily challenges, which become increasingly difficult due to the progressive nature of RP. Without an examination of individuals’ response to RP-related problems in their daily lives, it may be difficult to advise this population how to self-manage living with RP.

## Method

### Aim

This study aimed to explore the relationship between different types of coping strategies, emotional health, and VRQoL in individuals living with RP. This study investigated the following hypotheses: (H1) VRQoL will be higher in individuals with RP who use more engaging coping strategies compared to individuals with RP who use more disengaging coping strategies, (H2) emotional health will be higher in individuals with RP who use more engaging coping strategies compared to individuals with RP who use more disengaging coping strategies, and (H3) emotional health will be higher in individuals with RP who have higher VRQoL compared to individuals with RP who have lower VRQoL. The impact of emotional health on engaging and disengaging coping strategies was also explored.

### Design and setting

This study used a between-subjects, cross-sectional design using a questionnaire booklet. Three primary measures were established: type of coping strategy (Engaging Coping Strategies and Disengaging Coping Strategies; ECS and DCS respectively), Emotional Health, and VRQoL. Emotional Health was measured using Depression, Mental Wellbeing (MW), and General Happiness with Life (GHL), which were recorded using standardised questionnaires (detailed in materials). Only trait depression (that is, general depression and not state, or current, depression) was examined as this study was examining general Emotional Health. Emotional Health was included separately from VRQoL, as the VRQoL measurement used in this study only reflected Emotional Health in relation to vision. The aim of this study was to examine general Emotional Health, not merely Emotional Health in relation to vision. Thus, separate measures of Emotional Health were included. All participants completed the questionnaire booklet in their own time and preferred location.

### Participants

One hundred and seventy participants were recruited through the RP Fighting Blindness charity, UK. Sixty-five participants withdrew from the study, where no reason for withdrawal was given by the participants; as these 65 participants withdrew before completing the demographics, traits of the attrition population and the completion population could not be compared for significant individual differences. Of the remaining participants, 105 completed the study. Seventy were female, and the mean age was 46.98 (*SD* = 14.49, age range = 18–83). Eligible participants met two inclusion criteria: (1) at least 18 years of age and (2) they had been diagnosed with RP at least 1 year before participating in this study; this inclusion criterion was incorporated so that participants with RP had enough time to develop their coping strategy. There were no exclusion criteria.

### Materials

#### Coping Strategies Inventory – Short Form (CSI – SF)

This 32-item self-report questionnaire indicates different types of coping strategies that can be grouped into ECS and DCS. However, participants were not grouped into an ECS or DCS category. Each participant would have reported certain levels of both ECS and DCS, as evaluated in this study. This questionnaire has been validated and used in recent, primary research [12, 13].

#### Maryland’s Trait and State Depression Scale (MTSD)

This 36-item self-report questionnaire measures trait and state depression. Only the 18 items measuring trait depression were used, which examined general symptoms of depression. This self-reported questionnaire has been validated and used in recent, primary research.

#### The Warwick-Edinburgh Mental Wellbeing Scale (WEMWBS)

This 14-item self-report questionnaire measures MW. This questionnaire is reliable and has been validated [14, 15].

#### Subjective Happiness Scale (also known as General Happiness Scale, SHS)

This 4-item self-report questionnaire measures GHL. This questionnaire is reliable and has been validated [16].

#### National Eye Institute Visual Functioning Questionnaire 25 (NEI-VFQ 25)

There is no VRQoL measure that is specific for those with RP. Thus, the NEI-VFQ 25 was chosen due to its inclusiveness of a variety of vision-related daily tasks and its use in other degenerative eye conditions [17]. This 25-item self-report questionnaire measures VRQoL with the following subscales: General Health, General Vision, Ocular Pain, Near Activities, Distance Activities, Social Functioning, Mental Health, Role Difficulties, Dependency, Driving, Colour Vision, and Peripheral Vision. Role Difficulties is the perception of ability to accomplish goals or activities in relation to vision. Dependency is how dependent an individual is, where higher scores on Dependency indicated lower levels of dependency. This questionnaire is reliable and has been validated [17].

Age, sex, years since RP diagnosis (YSD), number of comorbidities (none, one or more than one; NC), participant-perceived RP severity (PPRPS), and knowing RP type (KRPT) were recorded as part of the demographics. Two versions of the questionnaire booklet were used for data collection: an online questionnaire and a Word document. The Word documents were for those participants who preferred this method of questionnaire completion and could be printed out by the participants. The online questionnaire was built using Qualtrics, and the document was built using Microsoft Word. Seven participants completed the Word document version, while 98 participants completed the Qualtrics online version. Due to a small number of participants who completed the Word document version compared to those who completed the online version, characteristics of these two groups were not compared for significant individual differences.

### Procedure

Questionnaires were organised into a booklet; this also included an information sheet, a consent form, a demographics form, and a debriefing sheet. Participants were recruited from the members of the UK RP Fighting Blindness charity. A letter of invitation was sent to potential participants with RP through the charity’s communication channels. The letter of invitation informed all recipients about the online version of the questionnaire booklet as well as the Word document version. This charity was chosen because it is the only charity specific to individuals who have RP, and therefore had access to a concentrated population of individuals that satisfies the inclusion criteria for this study. Thus, quota sampling was used to identify participants. Once participants read the information sheet and signed the consent form, they proceeded to complete the questionnaire booklet. If any participants could not sign or complete the questionnaire booklet due to their vision or any other condition, they were informed that they may have a trusted other (such as a carer or spouse) to complete the consent form and questionnaire booklet on their behalf. The trusted other was also asked to sign the consent form, to state that the participant is not directly completing the study. All information that was given to the participant was also available to the trusted other.

### Statistics and analysis

Please see the Additional file 1 for anonymised data. Demographic variables were controlled for in the analysis as previous studies have found these variables to influence coping strategies, Emotional Health, and VRQoL [4, 9, 18, 19]. Type of RP (recessive, dominant or X-linked) was also recorded, where 70 out of the 105 participants were not sure or did not know their type of RP. As subtype analysis could not be conducted due to the low numbers in each group (16 recessive, 11 dominant, 8 X-linked), participants were categorised into groups of knowing their RP type and not knowing their RP (35 knowing, 70 not knowing). The Driving subscale of the NEI-VFQ 25 was taken out as only 9 participants drove out of the 105 participants that completed the questionnaire booklet. Only the tertiary subgroups of the CSI-SF were used in the analysis, that is, ECS and DCS.

All primary measures (ECS and DCS, Emotional Health, and VRQoL) were analysed using a two-block hierarchical multiple regression model with the first block using the forward stepwise method and the second block using the enter method, as this model allows for controlling confounding variables of continuous data. All hierarchical multiple regressions controlled for age, sex, YSD, NC, PPRPS, and KRPT by inputting these variables into the first block. The following assumptions have been met for all hierarchical multiple regression analyses (see Table 1 for values): assumptions of independent errors with the Durbin-Watson values between 1 and 3 [20], assumptions of normal distribution with the Std Residual values between − 3.29 and 3.29 [21], assumptions of collinearity with the VIF (variation inflation factors) values less than 10 and Tolerance values greater than .1 [22], and assumptions of non-zero variances with all values above zero [23]. Assumptions of homogeneity of variance and linearity have also been met by all hierarchical multiple regression analysis. Assumptions of homogeneity of variance and linearity were analysed through scatterplots of standardised residuals, although scatterplots are not shown due to the number of hierarchical multiple linear regressions conducted. However, for assumptions of homogeneity of variance and linearity to be met, standardised residuals values of both the X and Y axes must be between − 3 and 3 [24], thus the assumption can also be seen from the aforementioned Std Residual values (Table 1).

**Table 1:** Minimum and maximum values of assumptions tests for all hierarchical multiple regressions

Test name	Minimum value	Maximum value
Durbin-Watson	1.68	2.47
Std Residuals	−2.74	2.70
VIF	0.71	3.35
Tolerance	0.30	1.00
Non-Zero Variance	45.16	933.837

Effect sizes and power calculations are reported as Cohen’s *f*^2^ and Power (1 – β) respectively [25]. Cronbach’s alpha was used to assess internal consistency for the standardized questionnaires. Cronbach’s alpha showed internal consistency was high and representative of population and questionnaire norms for all standardized questionnaires, CSI-SF α = .81; MTSD α = .96; WEMWBS α = .95; SHS α = .90; NEI-VFQ 25 α = .80.

## Results

### Participant traits

Participants had been diagnosed with RP for an average of 19.17 years (*SD* = 13.77, age range = 18–83) at the time of completing the questionnaire booklet. Sixty-five participants reported no co-morbidities, while 40 participants reported living with co-morbidities. Out of these 40 participants, 26 reported living with one co-morbidity, while 12 reported living with more than one co-morbidity. Two participants did not state their diagnosis of other condition(s). A Likert Scale was used to record PPRPS (1 = No Severity, 2 = Mild, 3 = Moderate, 4 = Severe, 5 = Extremely Severe) with a mean of 3.45 (*SD* = .76), where 88% of participants perceived their RP as being Moderately Severe to Severe (0% = No Severity, 10% = Mild, 45% = Moderate, 43% = Severe, 7% = Extremely Severe). Type of RP was also recorded, where 16 participants had recessive RP, 11 participants had dominant RP, and 8 participants had X-linked RP. Seventy participants did not know or were unsure of their type of RP; therefore, type of RP was excluded from further analysis. Participants were instead categorised into groups of knowing their RP type and not knowing their RP (35 knowing, 70 not knowing), and was analysed as a confounding variable.

### Primary measures

#### Testing H1

Coping strategies ECS and DCS were assessed on their predictive ability of VRQoL. Type of coping strategy significantly contributed to the prediction of General Health, Mental Health, Role Difficulties, and Dependency (Table 2). Type of coping strategy did not significantly contribute to the prediction of all other VRQoL variables, that is, visual functioning variables, ocular pain, and social functioning, and overall VRQoL (F Change *p* > .05). As the variance explained by ECS and DCS decreased from 10% to 8% for Role Difficulties, *f*^2^ value was negative, and thus no power calculation could be conducted. If *f*^2^ was positive, power (1 – β) would be .02. While this result is significant, the hypothetical *f*^2^ value indicates that there was little impact of ECS and DCS on Role Difficulties. However, it must be stated that this interpretation was based on a hypothetical result, and thus, it should be considered with caution. There was a stronger unique contribution of DCS to the prediction of all VRQoL subgroup variables compared to ECS. This indicates that DCS had a larger impact on VRQoL than ECS. There was a significant contribution of ECS only to Mental Health and Dependency; ECS did not significantly affect General Health and Role Difficulties. As ECS increased, Mental Health and Dependency increased. As DCS increased, General Health, Mental Health, and Dependency decreased, while Role Difficulties increased.

**Table 2:** Summary of hierarchical multiple regression of engaging and disengaging coping strategies contribution to predict VRQoL

Variable	β	*t*	R	R^2^	∆R^2^	F	Power (1 – β)	*f* ^2^
General health							.27	.03
Model 1			.27	.07	.07	7.36**		
NC^a^	−.27	−2.71**						
Model 2			.39	.15	.08	8.26***		
NC^a^	−.33	−3.39**						
Age	.28	2.93**						
Model 3			.49	.24	.09	7.45***		
NC^a^	−.32	−3.40**						
Age	.20	2.01*						
Engaging	.12	1.37						
Disengaging	−.29	−2.98**						
Mental health							.99	.37
Model 1			.22	.05	.05	4.78*		
PPRPS^b^	−.22	−2.19*						
Model 2			.34	.11	.07	6.09**		
PPRPS^b^	−.34	−3.17**						
YSD^c^	.28	2.67**						
Model 3			.65	.42	.30	16.52***		
PPRPS^b^	−.29	−3.26**						
YSD^c^	.17	1.89						
Engaging	.28	3.45**						
Disengaging	−.47	−5.70***						
Role difficulties							–	−.02
Model 1			.31	.10	.10	10.10*		
PPRPS^b^	.31	3.18**						
Model 2			.41	.17	.08	6.46*		
PPRPS^b^	.31	3.26**						
Engaging	−.02	−0.21						
Disengaging	.27	2.90**						
Dependency							.75	.09
Model 1			.29	.08	.08	8.81**		
PPRPS^b^	−.29	−2.97**						
Model 2			.49	.24	.16	9.95***		
PPRPS^b^	−.29	−3.21**						
Engaging	.19	2.11*						
Disengaging	−.33	− 3.71***						

*Note*. ^a^*NC* No. Of Comorbidities; ^b^*PPRPS* Participant-Perceived RP Severity, ^c^*YSD* Years Since (RP) Diagnosis

*p < .05, **p < .01, ***p < .001

There was a significant contribution of PPRPS to Mental Health, Role Difficulties, and Dependency. As PPRPS increased, Role Difficulties increased, while Mental Health and Dependency decreased. Age and NC significantly contributed to General Health; General Health increased with increasing age, and decreased with increasing NC.

#### Testing H2

Coping strategies ECS and DCS were assessed on their predictive ability of Emotional Health. Coping strategies significantly contributed to the prediction of all three variables of Emotional Health, that is, Depression, MW, and GHL (Table 3). There was a higher contribution of ECS to the prediction of MW compared to DCS. There was a higher contribution of DCS to the prediction of Depression compared to ECS. Both coping strategies similarly contributed to GHL. As ECS increased, MW and GHL increased, while Depression decreased. As DCS increased, Depression increased, while MW and GHL decreased.

**Table 3:** Summary of hierarchical multiple regression of engaging and disengaging coping strategies contribution to predict emotional health

Variable	β	*t*	R	R^2^	∆R^2^	F	Power (1 – β)	*f* ^2^
Depression							.99	.26
Model 1			.28	.08	.08	8.02**		
YSD^a^	−.28	−2.83**						
Model 2			.34	.12	.04	6.18**		
YSD^a^	−.28	−2.87**						
NC^b^	.20	2.02*						
Model 3			.39	.16	.04	5.76**		
YSD^a^	−.15	−1.34						
NC^b^	.25	2.53*						
Age	−.24	−2.12*						
Model 4			.44	.19	.04	5.51**		
YSD^a^	−.22	−1.89						
NC^b^	.22	2.23*						
Age	−.29	−2.53*						
PPRPS^c^	.22	2.04*						
Model 5			.68	.46	.27	12.89***		
YSD^a^	−.16	− 1.66						
NC^b^	.21	2.61*						
Age	−.13	−1.31						
PPRPS^c^	.13	1.38						
Engaging	−.18	−2.24*						
Disengaging	.51	6.07***						
Mental Wellbeing							.99	.25
Model 1			.286	.08	.08	8.57**		
YSC^a^	.29	2.93**						
Model 2			.588	.35	.26	16.60***		
YSD^a^	.24	2.81**						
Engaging	.42	4.98***						
Disengaging	−.28	−3.21**						
General Happiness with Life							.98	.20
Model 1			.23	.05	.05	5.10*		
Age	.23	2.26*						
Model 2			.32	.10	.05	5.40**		
Age	.33	3.07**						
PPRPS^c^	−.25	−2.34*						
Model 3			.38	.14	.04	5.20**		
Age	.36	3.42**						
PPRPS^c^	−.23	−2.13*						
NC^b^	−.21	− 2.10*						
Model 4			.42	.18	.04	5.05**		
Age	.34	3.28**						
PPRPS^c^	−.21	−2.04*						
NC^b^	−.22	− 2.22*						
Sex	−.19	−2.02*						
Model 5			.62	.39	.21	9.61***		
Age	.24	2.38*						
PPRPS^c^	−.17	−1.823						
NC^b^	−.20	−2.40*						
Sex	−.18	−2.19*						
Engaging	.31	3.74***						
Disengaging	−.33	−3.73***						

*Note*. ^a^*YSD* Years Since (RP) Diagnosis, ^b^*NC* No. of Comorbidities, ^c^*PPRPS* Participant-Perceived RP Severity

*p < .05, **p < .01, ***p < .001

There was a significant contribution of NC to the prediction of Depression and GHL; as NC increased, Depression increased, while GHL decreased. YSD significantly increased with MW. GHL significantly increased with Age. Females reported significantly higher levels of GHL compared to males.

#### Testing H3

Vision-related quality of life (VRQoL) was assessed on its predictive ability of Emotional Health (Table 4). Overall VRQoL did not significantly contribute to the prediction of Emotional Health (F Change *p* > .05); however, certain subgroup variables of VRQoL did significantly contribute to the prediction of Emotional Health. Due to the number of subgroup variables of VRQoL, only the significant variables within each model are shown in Table 4 (*p* < .05). Mental Health significantly contributed to the prediction of each variable of Emotional Health, with the biggest contribution to the prediction of GHL at 41%. As Mental Health increased, MW and GHL increased, while Depression decreased. As General Health increased, GHL increased, while Depression decreased. As Dependency increased, MW increased, while Depression decreased. Depression increased as Role Difficulties increased.

**Table 4:** Summary of hierarchical multiple regression of VRQoL contribution to predict emotional health

Variable	β	*t*	R	R^2^	∆R^2^	F	Power (1 – β)	*f* ^2^
Depression							.99	.52
Model 1			.31	.10	.10	9.84**		
YSD^a^	−.31	−3.14**						
Model 2			.38	.15	.05	7.68**		
YSD^a^	−.32	−3.25**						
NC^b^	.22	2.25*						
Model 3			.43	.19	.04	6.82***		
YSD^a^	−.19	−1.69						
NC^b^	.28	2.77**						
Age	−.25	−2.12*						
Model 4			.46	.23	.04	6.42***		
YSD^a^	−.26	−2.25*						
NC^b^	.24	2.40*						
Age	−.30	−2.57*						
PPRPS^c^	.23	2.11*						
Model 5			.80	.63	.41	8.87***		
YSD^a^	−.16	−1.75						
NC^b^	.20	2.42*						
Age	−.10	−0.97						
PPRPS^c^	−.05	−0.45						
General Health	−.23	−2.69**						
Mental Health	−.28	−2.69**						
Role Difficulties	.20	2.22*						
Dependency	−.27	−2.16*						
Mental Wellbeing							.99	.52
Model 1			.32	.10	.10	10.40**		
Age	.32	3.23**						
Model 2			.39	.15	.05	7.87**		
Age	.37	3.72***						
NC^b^	−.22	−2.21*						
Model 3			.75	.56	.41	7.73***		
Age	.18	1.89						
NC^b^	−.18	−2.05*						
Mental health	.30	2.74**						
Dependency	.35	2.62*						
General Happiness with Life							.99	.43
Model 1			.26	.07	.07	6.54*		
Age	.26	2.56*						
Model 2			.38	.14	.08	7.46**		
Age	.39	3.59**						
PPRPS^c^	−.30	−2.81**						
Model 3			.45	.20	.06	7.34***		
Age	.43	4.05***						
PPRPS^c^	−.27	−2.50*						
NC^b^	−.25	−2.50*						
Model 4			.49	.24	.04	6.94***		
Age	.41	3.93***						
PPRPS^c^	−.25	−2.42*						
NC^b^	−.26	− 2.65*						
Sex	−.21	−2.19*						
Model 5			.77	.59	.35	7.31***		
Age	.11	1.15						
PPRPS^c^	.03	.27						
NC^b^	−.19	−2.24*						
Sex	−.21	−2.54*						
General health	.33	3.61**						
Mental health	.41	3.70***						

*Note*. ^a^*YSD* Years Since (RP) Diagnosis, ^b^*NC* No. of Comorbidities, ^c^*PPRPS* Participant-Perceived RP Severity

*p < .05, **p < .01, ***p < .001

NC significantly contributed to the prediction of Emotional Health; as NC increased, Depression increased, and MW and GHL decreased. Females reported significant higher levels of GHL compared to males, as found when testing H2.

#### Investigating emotional health on ECS and DCS

Emotional Health was assessed on its predictive ability of ECS and DCS (Table 5). This association is the opposite direction of H2, and has been tested to better understand the relationship between Emotional Health and coping strategies. Emotional Health significantly contributed to the prediction of both ECS and DCS. Mental Wellbeing (MW) was the only variable that significantly predicted ECS, where ECS increased as MW increased. Depression was the only variable that predicted DCS, where DCS increased as Depression increased.

**Table 5:** Summary of hierarchical multiple regression of emotional health contribution to predict engaging and disengaging coping strategies

Variable	β	*t*	R	R^2^	∆R^2^	F	Power (1 – β)	*f* ^2^
Engaging							.99	.23
Model 1			.43	.19	.19	7.24***		
Depression	.06	0.56						
MW^a^	.42	2.91**						
GHL^b^	.062	0.44						
Disengaging							.99	.26
Model 1			.32	.10	.10	10.78**		
Age	−.32	−3.28**						
Model 2			.63	.39	.29	14.881***		
Age	−.17	−2.00*						
Depression	.50	4.94***						
MW^a^	.10	0.80						
GHL^b^	−.19	−1.49						

*Note*. ^a^*MW* Mental Wellbeing, ^b^*GHL* General Happiness with Life

*p < .05, **p < .01, ***p < .001

There was no impact of confounding variables for predicting ECS, indicating that Emotional Health made a significant contribution to predicting ECS with complete independence. Age was the only confounding variable that significantly contributed to DCS, where DCS decreased as age increased.

## Discussion

This study investigated the relationship between type of coping strategy (ECS and DCS), Emotional Health (Depression, MW, and GHL), and VRQoL in individuals with RP. Type of coping strategy did not impact overall VRQoL, but impacted four subgroups of VRQoL: General Health, Mental Health, Role Difficulties, and Dependency. Thus, coping strategies only influenced psychosocial aspects of vision-related problems. Specifically, ECS only influenced Mental Health and Dependency, while DCS influenced all four subgroups. Within Mental Health and Dependency, increased ECS was associated with increased Mental Health and Dependency, while increased DCS was associated with decreased Mental Health and Dependency. Therefore, H1 was partially supported; two psychosocial-related subgroups of VRQoL increased with ECS and not DCS. Both ECS and DCS impacted Emotional Health on all measures: Depression, MW, and GHL. DCS strongly impacted Depression compared to ECS, while ECS strongly impacted MW compared to DCS. An increase in ECS was associated with an increase in MW and GHL, and a decrease in Depression. The opposite with DCS was observed. Therefore, H2 was supported; Emotional Health increased with ECS, while it decreased with DCS. Emotional Health was not influenced by overall VRQoL but was largely influenced by four subgroups of VRQoL: Mental Health, General Health, Dependency, and Role Difficulties. Unsurprisingly, Mental Health was the only sub group of VRQoL that impacted all three measures of Emotional Health; higher scores of Mental Health were associated with increased MW and GHL, and decreased Depression. General Health only impacted GHL and Depression, where higher scores of General Health were associated with increased GHL and decreased Depression. Similarly, Dependency impacted MW and Depression, where increased Dependency was associated with increased MW and decreased Depression. Role Difficulties influenced Depression, where increased Role Difficulties was associated with increased Depression. Thus, H3 was only partially supported as Emotional Health increased with only four psychosocial-related subgroups of VRQoL. Examining the influence of Emotional Health on coping strategies, MW was the only measure of Emotional Health that influenced ECS, while Depression was the only measure of Emotional Health that influenced DCS. An increase in MW was associated with increased ECS, and an increase in Depression was associated with increased DCS.

These findings indicate that type of coping strategies influence psychosocial aspects of VRQoL and Emotional Health in individuals with RP. There is a positive influence of ECS and a negative influence of DCS on these factors. However, DCS influenced more VRQoL variables than ECS; this demonstrates that DCS has a larger negative impact on VRQoL than the positive impact ECS has on VRQoL. This indicates that DCS may be a stronger coping strategy than ECS; thus, reducing DCS may be more important than developing ECS in relation to VRQoL in those with RP. This is a surprising finding as previous research states that developing ECS improves VRQoL [9] or that ECS and DCS should be focused on together [18, 26]. This interpretation is further supported in this study, as the only measure of Emotional Health that the four VRQoL subgroups consistently influenced is Depression; Depression is the only Emotional Health measure that influenced DCS. Depression was also considerably more influenced by DCS than ECS. This suggests a cycle of maladaptive behaviour to manage RP that ultimately reduces VRQoL. Thus, reducing DCS may be more influential than developing ECS. Once DCS has been diminished, developing ECS may become more important; this study shows that ECS influences MW more than DCS, and that ECS improves psychosocial aspects of VRQoL, which is in concordance with previous evidence [9, 18, 26]. However, this result needs to be confirmed with further research, as although DCS influences more variables than ECS, prioritising DCS over ECS may not be appropriate.

It should be noted that majority of participants did not know their type of RP: whether it was recessive, dominant, or X-linked. This indicates that a large portion of individuals with RP in the general community do not have a full understanding of their visual condition. Trozzolino, Thompson, Tansman, and Azen [27] found that psychoeducation significantly improved management of visual impairment and emotional health suggesting that increasing awareness about RP should be developed within the general community, for example, by encouraging health professionals to give more information about RP to the relevant patients. However, knowing RP type did not seem to affect the relationship between VRQoL, Emotional Health, or type of coping strategy. This is in contrast to findings that suggest education about one’s own condition improves the management of that condition [27]. It may be that knowing type of RP does matter, or it may be that simply knowing type of RP is not enough. People with RP may need to understand the physiology of their condition as well as knowing their type of RP. However, it may also be that knowing the type of RP has no relationship to VRQoL, Emotional Health, or type of coping strategy. Future research must investigate this finding, as this may have implications on the management methods of RP in the general community.

The confounding variables recorded in this study (age, sex, YSD, NC and PPRPS; except knowing) were shown to be influential alongside the primary measures. In conjunction with the influence of ECS and DCS on VRQoL, NC and age influenced General Health, where General Health decreased as NC increased, and General Health increased as age increased. Mental Health, Role Difficulties, and Dependency (VRQoL variables) decreased as PPRPS increased. The positive correlation between age and General Health is noteworthy; it indicates that perception of general health improves as participants get older even though RP is a condition that gradually worsens over time. This is contradictory to pervious evidence, which states that decreased visual functioning is associated with decreased general health [28, 29]. However, this study found age to influence General Health alongside the primary measures, which was not conducted in other general vision studies. Therefore, this finding may be because individuals expect their vision to deteriorate over time, that is, they may get accustomed to their vision impairment. Eilertsen, Horgen, Kvikstad and Falkenberg [30] investigated indoor-lighting levels and perceived general health in 75 year-old individuals with vision impairments. They found that although indoor-lighting levels were below the standard recommendation, perceived general health was high, further displaying the possibility that individuals become accustomed to their vision impairment with age. Alongside the impact of coping strategies on Emotional Health, GHL increased as age increased, and MW increased as YSD increased. This further suggests that individuals with RP become accustomed to their condition as they grow older. Another possibility may be that their coping strategies change with age. Alongside the influence of Emotional Health on coping strategies, age was found to impact DCS with an increase in age associating with decreased DCS. This indicates individuals adjust to their RP with age and eventually decrease maladaptive behaviours.

Alongside the influence of coping strategies and VRQoL on Emotional Health, sex was found to impact the GHL measure; females had higher levels of GHL compared to males. This is an interesting outcome, as this study did not find any influence of sex on coping strategies, suggesting that there is no difference in type of coping used between females or males, whereas there is a difference in relation to Emotional Health. This indicates that the variables that account for the differences found between sexes in relation to Emotional Health was not recorded. A variable that may have affected this result is the support network status of each participant. Previous research indicates that females sought a stable support network more than males [31] and that a stable support network positively influences VRQoL [32, 33]. Thus, the status of an individual’s support network may have affected the results found in differences between females and males. However, other variables should also be considered, such as locus of control, self-efficacy, socioeconomic status or occupation.

The strength of this study is that the population is recruited from the general community; however, limitations should be considered to gauge the generalisability of the results. KRTP did not seem to affect the main measures of this study. This may be because knowing type of RP did not appear to make a difference on VRQoL, Emotional Health, and type of coping strategy. This is in contrast to the current literature, and needs to be investigated further. The measure used for VRQoL may have been too general for the specific population studied. This is because only psychosocial aspects of VRQoL were significantly impacted by coping strategies. Visual functioning aspects of VRQoL were not found to be significantly impacted by coping strategies, which is in contrast to previous evidence that shows this relationship [9, 34, 35]. This may be because the NEI-VFQ 25 was developed for general visual impairments; it was not exclusive to RP and its distinct symptoms [36]. Alternatively, it may be that coping strategies do not have any relationship with visual functioning within individuals with RP. However, since previous studies did find evidence to support this relationship, more examination should take place with a more specific VRQoL measure. This may also be the reason why this study found no effect of knowing RP type; it may be that the VRQoL measure was not suitable for the RP population. While it is suggested that an RP-orientated VRQoL be used in the future, there is currently no measure that has been developed. Thus, an RP-specific VRQoL measure should be first constructed for more accurate results of VRQoL. This study did not record whether participants were seeking treatment from a health professional or taking any medication at the time of the study. This may have altered the results if it had been taken into account as previous research has stated that treatments and therapies impact VRQoL [2, 7, 9]. Thus, future research aimed at a general community population should initially record treatment status and, if it is a longitudinal design, monitor any changes in treatment status.

Future research should focus on identifying factors that influence the relationship between VRQoL, coping strategies, and Emotional Health. This is because H1 and H3 were only partially supported, while H2 was supported. Future research should also examine the longitudinal effect of prioritising the reduction of DCS over the development of ECS in individuals with RP. Attention to the strength and length of this effect, and at what stage this effect is seen (initial or other stages of an intervention) should take precedence to fully understand this phenomenon. Stevelink and Fear [37] found that visual impairment negatively affects psychosocial wellbeing initially, but participants learnt to cope with it over time, suggesting that reducing DCS initially might be more beneficial to the VRQoL in individuals with RP. This may be because recognising and reducing DCS will allow participants room for founding and developing ECS later on. The effects of age on coping strategies and VRQoL in individuals with RP should be investigated. Even though RP is a degenerative condition, results of this study show that DCS decreased when perception of general health increased. Thus, the mechanism behind this phenomenon should be examined through qualitative analysis, through either a retrospective cross-sectional design or a prospective longitudinal design. More examination on the effects of sex should also be conducted. This study found that there was a difference between females and males in relation to GHL, however, no difference in coping strategies was found between females and males. Thus, other variables should be taken into account, such as support network status, to investigate the disparity in Emotional Health within sex. Furthermore, future research may conduct a case-controlled, mixed methods (qualitative and quantitative) study that includes individuals with RP and individuals without RP to compare coping strategies, VRQoL and Emotional Health. This will allow for a deeper understanding of the issues individuals with RP face compared to individuals without RP as well as examining the generalisation of the findings from this study.

## Conclusion

The findings from this study highlight the importance of type of coping strategies and their influence on psychosocial aspects of VRQoL and Emotional Health in individuals living with RP. Additionally, it reflects the various factors that may aid in the adaptation to living with RP, and the various factors that still need to be identified. Coping strategy findings may help inform further research towards examining effective methods for reducing DCS to achieve positive adaptation to RP-related problems, which may lead to improved VRQoL in those living with RP.

## Additional file

Additional file 1: Anonymised Survey Data. (XLSX 10 kb)

## Abbreviations

CSI – SF: Coping Strategies Inventory - Short Form
DCS: Disengaging coping strategies
ECS: Engaging coping strategies
GHL: General Happiness with Life
KPRT: Knowing RP type
MTSD: Maryland’s Trait and State Depression Scale
MW: Mental Wellbeing
NC: Number of comorbidities
NEI-VFQ 25: National Eye Institute Visual Functioning Questionnaire 25
PPRPS: Participant-Perceived RP Severity
RP: Retinitis pigmentosa
SHS: Subjective Happiness Scale
VRQoL: Vision-related quality of life
WEMWBS: Warwick-Edinburgh Mental Wellbeing Scale
YSD: Years Since (RP) Diagnosis
